# Numerical model of hybrid mode-locked Tm-doped all-fibre laser

**DOI:** 10.1038/s41598-020-74194-7

**Published:** 2020-10-15

**Authors:** Aleksandr Donodin, Vasilii Voropaev, Daniil Batov, Dmitrii Vlasov, Vladimir Lazarev, Mikhail Tarabrin, Aleksandr Khegai, Mikhail Likhachev

**Affiliations:** 1 Aston Institute of Photonic Technologies, Aston University, Birmingham, B4 7ET UK; 2 Science and Education Center for Photonics and IR-Technology, Bauman Moscow State Technical University, Moscow, 105005 Russia; 3 Frequency Standards Laboratory, P. N. Lebedev Physical Institute of the Russian Academy of Sciences, Moscow, 119991 Russia; 4 Dianov Fiber Optics Research Center, Prokhorov General Physics Institute of the Russian Academy of Sciences, Moscow, 119333 Russia

**Keywords:** Fibre lasers, Mode-locked lasers, Ultrafast lasers

## Abstract

Ultrafast Tm-doped fibre lasers have been actively studied for the last decade due to their potential applications in precise mid-IR spectroscopy, LIDARs, material processing and more. The majority of research papers is devoted to the comparison between a numerical modelling and experimental results; however, little attention is being paid to the comprehensive description of the mathematical models and parameters of the active and passive components forming cavities of Tm-doped all-fibre lasers. Thus, here we report a numerical model of a stretched-pulsed Tm-doped fibre laser with hybrid mode-locking and compare it with experimental results. The key feature of the developed numerical model is employment of the experimentally measured dispersion coefficients and optimisation of some model parameters, such as the bandwidth of the spectral filter spectral filtering and the saturation power of the active fibre, for a conformity with the experiment. The developed laser emits 331.7 fs pulses with a 23.8 MHz repetition rate, 6 mW of average power, 0.25 nJ of pulse energy, and a 21.66 nm spectral bandwidth at a peak wavelength of 1899.5 nm. The numerical model characteristics coincide with experimentally achieved spectral width, pulse duration, and average power with inaccuracy of 4.7%, 5.4%, and 22.9%, respectively. Moreover, in the discussion of the work the main possible reasons influencing this inaccuracy are highlighted. Elimination of those factors might allow to increase accuracy even more. We show that numerical model has a good agreement with the experiment and can be used for development of ultrafast Tm-doped fibre laser systems.

## Introduction

Mode-locked lasers based on Tm-doped fibres have been intensively studied in the last decade due to a high demand on sources emitting ultrashort pulses at the wavelengths of around $2\,\upmu {\text {m}}$ for a wide variety of applications, such as vibrational spectroscopy for medicine, environmental sensing and supercontinuum generation^1,2^. The short pulse duration (less than 150 fs), the sufficient peak power, and the stable generation regime are the essential parameters of a laser for the broadband and coherent supercontinuum generation in highly nonlinear fibres^3^.

The diversity of mode-locking mechanisms with high performance in the desired spectral region around $2\,\upmu {\text {m}}$—such as nonlinear polarisation evolution (NPE)^4–6^, nonlinear loop mirror^7,8^, single wall carbon nanotubes (SWCNT)^9–11^, ${\text {Bi}}_2 {\text {Te}}_3$^12^, SESAM^13–15^, ${\text {MoS}}_2$^16^, graphene^17^—allows to achieve ultrashort pulses with required duration, power level along with high stability and regime repeatability. A huge variety of generation regimes were achieved in Tm-doped fibre systems, such as: soliton^7,10,11,15,17–21^, stretched pulse^5,6,9,10,15,22^, dissipative soliton^4,8–10,13,15,23,24^, noise-like pulse^14,23,25^ and bound solitons^12,16,26^. Soliton Tm-doped fibre lasers have relatively narrow spectral width and, thus, long pulse duration compared with stretched-pulse lasers. Solitons with relatively short pulse duration (190 fs) were achieved in the Tm-doped fibre laser with small absolute value of group delay dispersion (GDD)^27^; however, the achieved pulse energy is only 20 pJ. Use of fibres with normal group velocity dispersion (GVD) (e.g. germanosilicate fibres with small core diameter (less than $5\,\upmu {\text {m}}$ ) and high concentration of germanium (more than 20%)) or bulk diffraction grating based compressors allow to improve the performance of Tm-doped fibre systems at $2\,\upmu {\text {m}}$ via dispersion management^28^. The dispersion management allows to achieve pulse energies of more than 2 nJ^22,29^.

The numerical modelling has always been a great tool for the design of mode-locked lasers and the pulse evolution analysis. Various numerical models based on different modifications of the nonlinear Schrödinger equation for simulation of intracavity pulse propagation were proposed^30–33^ and implemented^34–37^ to study ultrafast lasers during last three decades. The majority of these papers were focused on studying of Er- and Yb-doped fibre lasers because of the current relative maturity of these laser sources. However, the numerical modelling of Tm-doped ultrafast laser sources is of the current interest and was presented in^20,21,29,38–41^. Despite the comprehensive analysis of generation regimes and the comparison between the numerical modelling and the experimental results, little attention is being paid to the detailed description of the used mathematical model and the parameters of active and passive components of cavity (GVDs of fibres, nonlinear coefficients of fibres, spectral characteristics of the laser cavity components, parameters of the active fibres, parameters of SAs, etc.).

Thus, in this work we present the numerical model of the stretched-pulse Tm-doped all-fibre laser with hybrid mode-locking that is verified by the experimental data. The numerical simulation based on the nonlinear Schrödinger equation solved by split-step Fourier method is used. Moreover, observation of the intracavity pulse evolution is conducted for the analysis of the regime performance. The stretched-pulse generation is achieved by obtaining slightly anomalous GDD using normal GVD fibres with high the germanium oxide concentration (30%) and the high numerical aperture for both dispersion management and total cavity nonlinearity magnification. The SWCNT saturable absorber and the NPE technique are chosen as the mode-locking mechanisms. Based on our experience, the use of a polariser in a laser cavity providing the NPE mechanism allows to prevent the generation of a continuous wave (CW) component along with ultrashort pulses, leading to a more efficient subsequent amplification of the pulses. The parameters of the spectral filter and active fibre are varied to correspond to the experiment results. The discrepancy of the pulse duration and the width of the spectrum obtained in the model and the experiment is 5.4% and 4.7%, respectively. The error of the modelled value of the output power is 22.9%. A potential reason for these discrepancies are discussed in the paper.

The remainder of this paper is organised as follows: Section 2 describes the laser system and gives a description of the theoretical model along with the parameters of the cavity components used in the numerical simulation. Section 3 describes the results achieved both numerically and experimentally and shows the dynamics of intracavity pulse evolution. In Section 4 the reasons for discrepancies between experimental and numerical results are discussed, and possible solutions to avoid errors are suggested.

## Laser setup and numerical model

**Figure 1:**
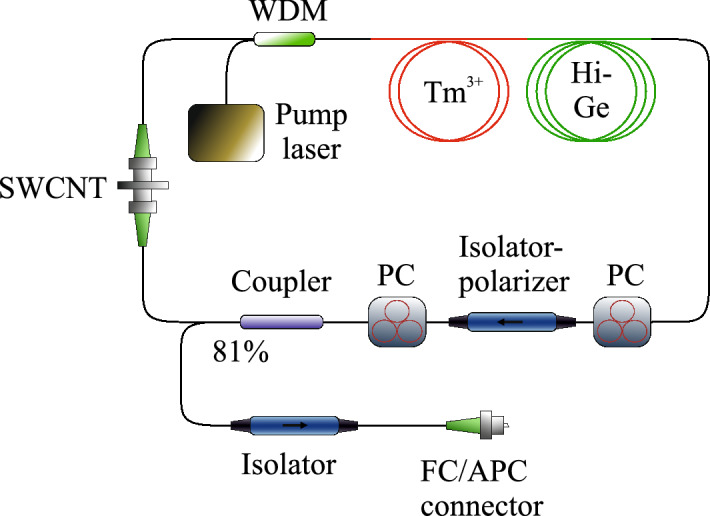
Scheme of the thulium-doped fibre ring laser with hybrid mode-locking, WDM: wavelength-division multiplexer, PC: polarisation controller, ${\text {Tm}}^{3+}$: thulium-doped fibre, Hi–Ge: fibre with high concentration of germainum oxide, SWCNT: single wall carbon nanotubes.

**Figure 2:**
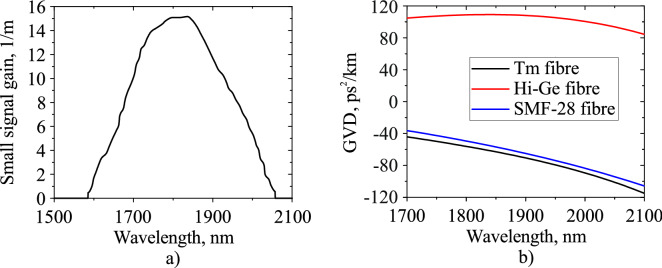
(**a**) Small signal gain of the active Tm-doped fibre; (**b**) group velocity dispersion (GVD) of cavity fibres.

Firstly, we describe the setup of the developed ultrafast Tm-doped all-fibre laser. Figure 1 shows a scheme of the thulium-doped all-fibre ring laser with hybrid mode-locking. The radiation of a CW erbium/ytterbium-co-doped fibre laser operating at a wavelength of 1550 nm is coupled with wavelength-division multiplexer (WDM) and comes through a segment of the step-index ($\Delta {\text {n}}=0.012$, core diameter ${{\text {d}}}_{\mathrm{c}}= 10\,\upmu {\text {m}}$) thulium-doped aluminosilicate (0.8 wt% thulium, 3.6 wt% aluminum) glass fibre with anomalous GVD of $-\,70.56\,{{\text {ps}}}^{2}/{\text {km}}$ at 1900 nm, where it is dramatically absorbed (more than 98%). Small signal gain is shown in Fig. 2a. The form of the small signal gain was taken from the consideration of its similarity with emission cross section^42^.

An isolator-polariser is used for unidirectional generation and in combination with two polarisation controllers (PCs) enables the NPE technique which works as the mode-locking mechanism together with the saturable absorber based on the polymer film with SWCNT located between two FC/APC connectors. An output coupler removes 81% of the total power from the cavity and has 5% of total insertion loss. An additional isolator on the output is used to prevent the beam to reflect back to the cavity. An FC/APC connector is used for convenient connection with measurement instruments. The additional fibre with the high concentration of germanium (30% germanium oxide, core diameter ${{\text {d}}}_{\mathrm{c}}=2.2\,\upmu {\text {m}}$) (Hi–Ge) with a GVD value of $108\,{{\text {ps}}}^{2}/{\text {km}}$ and nonlinear coefficient of 3.9 1/W/km is used for dispersion management and makes GDD slightly anomalous. It further helps to achieve the non-solitonic behaviour of the pulse. The dependencies of the cavity fibres dispersion parameters on the wavelength are depicted in Fig. 2b. The dispersion of the fibres was measured using the setup described in^43^. In this section we present a general laser scheme, all exact fibre lengths are presented in the next section. The cavity GDD value is ${-}1.74 \times 10^{-3}\,{\hbox {ps}}^{2}$. The numerical model is based on the modified nonlinear Schrödinger equation and can be represented as a consecutive propagation through the cavity elements. The split-step Fourier method is used to solve the modified nonlinear Schrödinger equation for passive and active fibres^31^:

1 $$\begin{aligned}&\frac{\delta A(z,T)}{\delta z} +\frac{\alpha }{2}A(z,T)-\sum _{k\ge 2}\beta _k\frac{i^{k+1}}{k!}\frac{\delta ^k A(z,T)}{\delta T^k} = i\gamma |A(z,T)|^2 A(z,T), \end{aligned}$$

2 $$\begin{aligned}&\frac{\delta A(z,T)}{\delta z} +\frac{\alpha }{2}A(z,T)-\frac{g}{2}A(z,T)-\sum _{k\ge 2}\beta _k\frac{i^{k+1}}{k!}\frac{\delta ^k A(z,T)}{\delta T^k} = i\gamma |A(z,T)|^2 A(z,T). \end{aligned}$$

Here *A*(*z*, *T*) is a slowly varying complex envelope of the pulse propagating through fibre with length z and time $T = t-\beta _1z$, which represents the moving frame in real time *t* with an envelope group velocity $\beta _1^{-1}$. Moreover, the amplitude is normalised such that $|A(z,T)|^2$ gives the instantaneous power in watts. $\beta _k$ are the parameters of $k^{th}$ order of dispersion, $\gamma$ is the nonlinear coefficient, $\alpha$ is the attenuation constant, and *g* is the gain which spectral dependence is included in the frequency domain through the following parameter^34^:

3 $$\begin{aligned} {\hat{g}}(\lambda ) = g_0(\lambda )\cdot \frac{1}{1+\frac{E_0}{E_{sat}}}, \end{aligned}$$

where $g_0$ is the small signal gain which is depicted in Fig. 2a, $E_{sat}$ is the saturation energy, which is defined through the saturation power and the repetition frequency as $E_{sat} = P_{sat}/f_{rep}$, and the total pulse energy $E_0$, defined as an integral of a field envelope over the time $E_0 = \int _{ }{ }|A|^2dT$. All the parameters of the passive and active fibres used in the numerical model, including dispersion coefficients at the wavelength of 1900 nm, the loss or gain characteristics, are shown in Table 1. The nonlinear coefficients were calculated using the formula $\gamma = \omega _0 n_2/(c A_{eff})$^31^, where $\omega _0$ is the central frequency, $n_2$ is the nonlinear refractive index and is used from the paper^44^, *c* is the speed of light, and $A_{eff}$ is the effective mode area and is calculated from the fibre V parameter^45,46^.

**Table 1:** Active and passive fibre characteristics.

Waveguide	SMF-28 waveguide	${\text {Tm}}^{3+}$ Waveguide	Hi–Ge fibre
$\beta _2$ (${{\text {ps}}}^{2}$/km)	− 65	− 70.56	108
$\beta _3$ (${{\text {ps}}}^{3}$/km)	0.325	0.311	0.07
$\beta _4$ (${{\text {ps}}}^{4}$/km)	$-1.8\times 10^{-3}$	$-2.1\times 10^{-3}$	$-2.6\times 10^{-3}$
$\beta _5$ (${{\text {ps}}}^{5}$/km)	$1.28\times 10^{-5}$	$2.68\times 10^{-5}$	$3.1\times 10^{-5}$
$\gamma$ (1/W/km)	0.53	1.02	3.9
$\alpha$ (1/m)	$4.6\times 10^{-3}$	$4.6\times 10^{-3}$	$4.6\times 10^{-3}$
$g_{0max}$ (1/m)	–	15	–
$P_{sat}$ (mW)	–	Variable	–

To estimate the transmission functions of the saturable absorbers (SA) in the time domain the following equation was used:

4 $$\begin{aligned} K(T) = 1-[q_{sat}+q(T,|A(T)|^2)], \end{aligned}$$

where ${q_{sat}}$ is a saturated losses of a SA, and ${q(T,|A(T)|^2)}$ is a function of saturation of a SA. For the SWCNT SA the following differential equation for a slow SA^47^ is solved with the $4^{th}$-order Runge-Kutta method^48^:

5 $$\begin{aligned} \frac{\delta q(T,|A(T)|^2)}{\delta t} = -\frac{q(T,|A(T)|^2)-(q_{sat}-q_{uns})}{\tau }-q(T,|A(T)|^2)\frac{|A(T)|^2}{P_{abs}}. \end{aligned}$$

Here ${q_{uns}}$ and ${q_{sat}}$ are the levels of unsaturated and saturated losses taken from the insert of Fig. 9 in^49^, $\tau$ is the relaxation time of the SA taken from Fig. 7 in^49^ and $P_{abs}$ is the saturation power of the SA calculated from the insert in Fig. 9 in^49^. The similar characteristics of carbon nanotubes with polymer film base are reported in^50,51^. The unsaturated loss here are referred to the maximum total losses of SA in the case of the low incident signal power. In the case of the NPE SA with relatively small relaxation time the equation (5) can be simplified to the equation^52^:

6 $$\begin{aligned} q(T,|A(T)|^2) = \frac{q_{uns}-q_{sat}}{1+\frac{|A(T)|^2}{P_{abs}}}. \end{aligned}$$

Moreover, to introduce the spectral selectivity of the cavity components the spectral filter with Gaussian shape with the spectral bandwidth $\Omega$ and spectral shift of the filter $\omega _0$ is included in the model after NPE SA:

7 $$\begin{aligned} K(\omega ) = \exp {\left( -\frac{(\omega -\omega _0)^2}{2\Omega ^2}\right) }. \end{aligned}$$

**Table 2:** Parameters of passive cavity components.

Element	Parameter	Value	Element	Parameter	Value
SWCNT^49^	Modulation depth	0.33	NPE	Saturation power (W)	1000
	Saturation power (W)	10.05		Modulation depth	0.1
	Unsaturated loss	0.65	Output coupler	Coupling ratio	0.14/0.81
	Recovery time (ps)	0.37	Spectral filter	Filter bandwidth (nm)	Variable
				Filter spectral shift (nm)	Variable

The coupler is described by the simple transmission function $Q=(1-R_{out}-\alpha )$, where $R_{out}$ is the output coupling efficiency and $\alpha$ is the level of the internal loss of the coupler equal to 5%. The parameters of the passive components used in the model are depicted in Table 2. The achieved splice loss between fibres with different mode-fields (e.g. the splice between the Hi–Ge fibre and both Tm-doped and SMF fibres) are experimentally measured and are approximately equal to 0.63 dB.

## Experimental and numerical results

**Figure 3:**
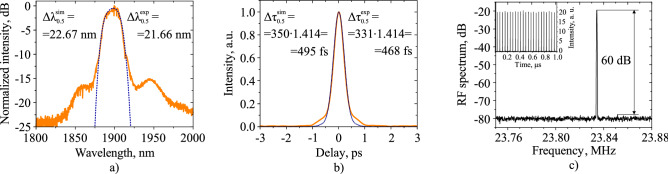
(**a**) Spectrum; (**b**) intensity autocorrelation trace; orange curves represent experimental data, blue dotted curves represent results of numerical simulation; (**c**) radio-frequency spectrum of achieved pulses; inset shows pulse train captured by oscilloscope.

The stable mode-locking regime of the experimental setup is achieved by adjusting PCs at the incident pump power of 350 mW with an average output power of 6 mW. The measured characteristics are shown in Fig. 3. The achieved spectrum is shown in Fig. 3a (orange curve), the centre wavelength is 1899.5 nm, the spectral full width at half maximum (FWHM) is 21.66 nm. There are small side wings that are present in both time and spectral domains, and the possible reasons for their appearance are discussed in the last section. The experimentally achieved autocorrelation trace is depicted in Fig. 3b (orange curve) and has Gaussian shape with FWHM of 469 fs, that correspond to 331.7 fs FWHM of pulse. The pulse energy is equal to 0.25 nJ. However, as the time-bandwidth product (TBP) for bandwidth-limited Gaussian pulse is $\approx \,0.44$^53,54^ and the TBP for achieved pulse is equal to 0.595, the pulse has a chirp. Figure 3c shows the radio frequency spectrum, repetition rate of the pulses is 23.835 MHz and the signal-to-noise ratio is 60 dB. The insert in Fig. 3c shows the captured oscilloscope pulse trace with the amplitude modulation with a value of ± 2.85% relative to the average value.

**Figure 4:**
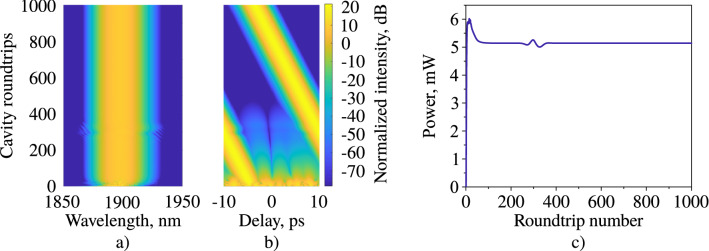
(**a**) Spectral, (**b**) temporal, (**c**) average power evolution on the roundtrip at the output coupler.

Afterwords, the parameters of the spectral filter and the saturation power of the active fibre in the numerical model are manually varied that the spectrum, the average power, and the autocorrelation trace at the output of the FC/APC connector coincide most accurately with the measured values. The input field of the model is represented as the one-photon-per-mode noise which is amplified in the active fibre. The filter spectral bandwidth ($\Omega$) and the filter centre wavelength appears to be 5.63 rad × THz (10.8 nm), and 1901 nm, respectively, and the active fibre saturation power is 0.93 mW. In this particular model the total gain varies through the saturation power and, thus, defines the average power^34^. A stable generation regime with a time window size of 20 ps and the number of the grid points of ${2^{11}}$ is achieved. It should be noted that the temporal and spectral resolutions of this numerical model is 10 fs and 50 GHz, respectively, however, during the analysis of the output power distributions in both spectral and temporal domain, they were interpolated with an increased resolution (in 100 times) leading to the decreased temporal and frequency grid step to 0.1 fs and 0.05 GHz. The increase of grid points to ${2^{13}}$ in time domain with the same bandwidth of the time window led to a relative change of pulse and spectral width less than 0.04%, while the computation time was increased by 3.7 times. The results of pulse generation in the designed laser scheme is presented in Fig. 4. This figure shows the spectral (a) and temporal (b) distributions of the intensity and the average power (c) at the laser output.

The convergence criterion is described in^37^, and in our case the relative change $\epsilon$ should be less $1.2\cdot {10^{-6}}$ for at least 300 roundtrips. Thus, the stable pulse generation starts from the 430th roundtrip. The temporal shift of the pulse in the time window corresponds to the displacement of the central wavelength from 1900 nm (centre of the frequency window), and misalignment between the speed of the pulse and speed of the time frame due to nonzero dispersion parameter^31^. The fibre lengths before measurement devices are also included in the model to directly compare the autocorrelation traces and spectra on the output of the experimental setup and theoretical model. The total length of SMF-28 at the laser output is 2.3 m, and contains an optical isolator with the total internal loss of 0.45 dB. The modeled average laser output power in the stable generation regime is 4.63 mW after the output isolator. The numerical autocorrelation trace and spectrum match with a high precision (Fig. 3a, b), the achieved pulse duration is 349.61 fs, the achieved spectrum FWHM is 22.67 nm.

**Figure 5:**
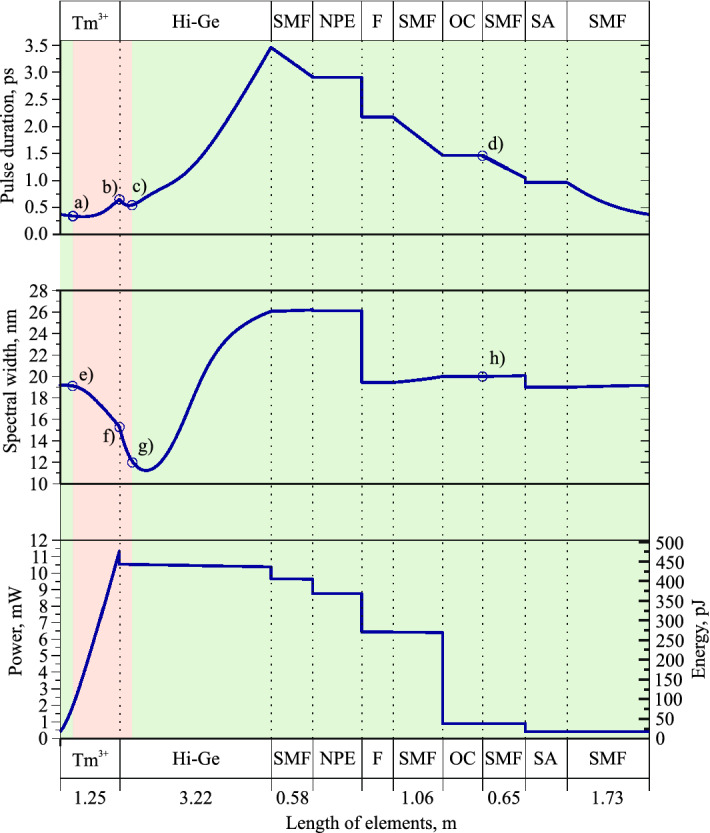
The intracavity pulse performance based on numerical simulations.

Figure 5 shows the evolution of the three main pulse parameters as functions of the position in the cavity—the pulse duration, the spectral width, and the average power—and illustrates the principle characteristics of the laser operation. At the top of the figure there is a schematic of the laser which consists of the Tm-doped fibre (${\text {Tm}}^{3+}$), the nonlinear fibre with the high concentration of germanium (Hi–Ge), the standard telecommunication SMF-28 fibre, the optical isolator-polariser which enables the nonlinear polarisation evolution technique as the saturable absorber (NPE), the filter (F), the output coupler (OC), and the SWCNT as the saturable absorber (SA). Here we assume that the filter depicts spectral dependencies of all cavity components except fibre gain whose spectral dependency is included in the gain function; however, it is placed just after the NPE because this technique introduces the most of spectral selectivity in the cavity^55–57^. The length of the finite elements (optical fibres) are shown in the horizontal axis of Fig. 5. The background colour depends on the pulse chirp: the red one refers to the negative chirp sign, and the green one refers to the positive chirp sign.

**Figure 6:**
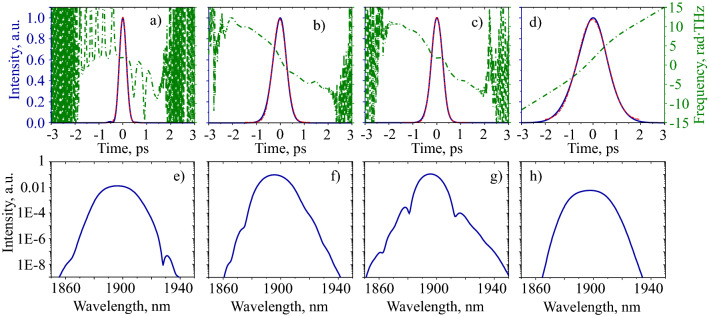
Temporal and spectral distributions of pulses and chirps inside the cavity in the following points: the chirp sign change (from positive to negative) in the Tm-doped fibre (**a**, **e**), the end of Tm-doped fibre (**b**, **f**), the chirp sign change (from negative to positive) in Hi–Ge fibre (**c**, **g**), the output coupler (**d**, **h**). Blue lines are the results of the numerical modelling; red dots are the approximation using a Gaussian shape function; green lines and dots show the chirp distributions in time.

In the first 30 cm of fibre, the pulse undergoes the amplification, the spectral and temporal narrowing along with the negative chirp obtainment in the active fibre. After that the chirp of the pulse changes the sign. The temporal and spectral distributions at this point are shown in Fig. 6a, e, respectively. The temporal profiles of the pulse at all points are approximated using a Gaussian function. After that the pulse spectrum is narrowed along with temporal broadening until the end of the fibre. Temporal and spectral distributions at the end of the active fibre are shown in Fig. 6b, f, respectively. Afterwords, the pulse goes through Hi–Ge fibre. The length of this fibre is large enough to change the sign of the pulse chirp back to normal and enable the mechanism of spectral and temporal broadening together. The temporal and spectral distributions at the second zero-chirp point are shown in Fig. 6c, g. Then the pulse passes through a cascade of two SAs, filter and OC connected with each other by SMF-28 fibre. The additional cavity point is shown in Fig. 6: pulse at the output of the laser (d,h). On this basis, pulse dynamics proves the assumption about the stretched-pulse generation regime^35^, as there are the pulse duration has 2 local maximums and local minimums, and the chirp changes the sign 2 times in the cavity.

The maximum spectral width of the generation regime is mostly determined by the interplay between the nonlinear processes and the normal dispersion that occurs in the Hi–Ge fibre. However, the optical filter partially limits the pulse spectrum. Even though the SAs do not determine pulse duration, the minimal pulse duration is defined by evolution in both active and passive fibres and the interplay between the pulse and spectral broadening in the Hi–Ge fibre. Moreover, the NPE technique contributes little in the pulse shaping, and only includes high spectral selectivity into the cavity, as the influence of the filter changes both spectral and temporal widths dramatically. The pulse compression factor, which is the ratio between the maximum and minimum pulse duration in the cavity, is approximately 10, thus the main pulse dynamics mechanism is the pulse breathing^58^ that arises due to the dispersion map and the nonlinear pulse evolution. The nonlinear phase (B-integral) in the cavity is 1.69 $\pi$, which is consistent with the value for the stretched-pulse generation regime^59^.

The achieved experimental results and the direct comparison with the numerical modelling show that the developed numerical model is accurate enough to predict output characteristics of the generation regime of ultrafast Tm-doped fibre lasers. Moreover, the presented numerical model has proved to be a good tool for the analysis of generation regimes in Tm-doped laser, as it shows intracavity evolution of pulse characteristics e.g. both spectral and temporal width and shape, as well as chirp and power distributions.

## Discussion

**Figure 7:**
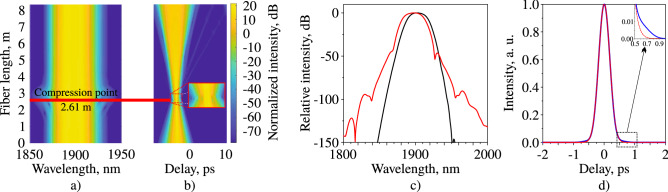
(**a**) Spectral and (**b**) temporal pulse evolution on the output SMF-28 fibre length, spectrum at the laser output (**c**, black curve), spectrum at the compression point (**c**, red curve), autocorrelation trace at the compression point (**d**, blue curve), Gaussian fit (**d**, red dotted curve).

In this section we want to briefly discuss discrepancies between the model and the experiment and the possible reasons for that behavior. Compared to the theoretical data the measured pulse autocorrelation has small side wings, and there are side peaks in the measured spectrum. In addition, the measured output power (6 mW) is more than the theoretical one (4.7 mW). Moreover, the pulse train has the small amplitude modulations that are not predicted in the model. Since the energy of the pulse turned out to be larger in the experiment than in the model it can be assumed that the appearance of side wings and peaks in the autocorrelation and in the spectrum is associated with the nonlinear deformation of the pulse in both time and spectral domains in the measurement line. Figure 7 shows the propagation of the laser pulse achieved in the numerical modelling in the extended measurement fibre in spectral (a) and temporal (b) domain.

The comparison of the spectrum at the laser output and at the compression point (2.6 m) is shown in Fig. 7c. The autocorrelation trace of the pulse at the compression point with a Gaussian approximation is shown in Fig. 7d. The spectrum undergoes the broadening at the compression point and the small side wings appear in the time domain. The inset of Fig. 7d shows that in comparison to the Gaussian approximation there are lateral wings in the autocorrelation trace of the pulse in the compression point. We assume that all the above disparity between the model and the experiment are related to the following assumptions accepted in the model. Firstly, we use an optimised Gaussian filter in the cavity, despite the fact that each component of the cavity introduces its own spectral filtering to the pulse. Secondly, we presented a simplified model of the active medium; however, the laser rate equations are more accurate. Thirdly, errors in parameters of the cavity components can affect discrepancies, including the dispersion coefficients and the nonlinear coefficients of all fibres, parameters of SWCNT, etc. Finally, we neglect the birefringence and the effect of stimulated Raman scattering in media. Despite these assumptions the pulse behavior in time and spectral domains in the model and in the experiment has very good agreement. Analysis of the influence of the above mentioned effects on the pulse dynamics inside and beyond the cavity is might be a possible solution for accuracy increase for the problems of modelling of more complex Tm-doped fibre systems.

## Conclusion

In this paper the numerical model of an all-fibre Tm-doped ultrafast laser is presented and experimentally verified. The generated pulses have the 331.7 fs duration and the 21.66  nm spectral bandwidth. The evolution of the pulse within the cavity was studied with the adapted numerical model and showed vast spectral and temporal breathing during the pulse propagation which corresponds to the stretched-pulse generation regime. The developed numerical model has good agreement with the experimental results and can be used as the robust and versatile instrument for the Tm-doped fibre laser design and the pulse generation regime analysis. Discrepancies between the model and the experiment were discussed. The developed laser setup is a first step towards the system of supercontinuum generation in the mid-IR region.
